# Impact of E-Coach chronic disease management model combined with the WeChat platform on self-management ability in hypertension patients

**DOI:** 10.3389/fcvm.2026.1707441

**Published:** 2026-07-06

**Authors:** Xianzhi Lv, Pinnan Zhao, Junxi Chen, Min Li

**Affiliations:** 1 Health Monitoring Department, Chengdu High-tech Guixi Community Healthcare Center, Chengdu, China; 2 Health Monitoring Department, Chengdu High-tech Zone Center for Disease Control and Prevention, Chengdu, China; 3 Endemic Disease Prevention and Control Department, Chengdu Jintang Municipal Center for Disease Control and Prevention, Chengdu, China

**Keywords:** E-Coach chronic disease management model, hypertension, patient-perceived chronic illness care, self-management, WeChat platform

## Abstract

**Objective:**

Hypertension remains a major global health challenge requiring effective long-term management. This study evaluated the impact of the E-Coach chronic disease management model integrated with WeChat on self-management capabilities in patients with hypertension.

**Methods:**

This was a single-center randomized controlled trial. A total of 250 middle-aged and elderly hypertension patients were randomly divided into either a control group (conventional hypertension management) or an intervention group (comprehensive management based on the E-Coach chronic disease model delivered via the WeChat platform) (*n* = 125). The primary outcomes included Chronic Disease Self-Management Study Measures (CDSMS) scores and patient-perceived chronic illness care assessed using Patient Assessment of Chronic Illness Care (PACIC) scores. The secondary outcomes included systolic blood pressure (SBP) and diastolic blood pressure (DBP), management outcomes (e.g., hospital visits, medication adherence, blood pressure (BP) control rates.

**Results:**

After six months, CDSMS scores across all dimensions (e.g., stretching and strength exercises [45 (15, 45) vs. 45 (15, 45)], aerobic exercises [120 (120, 180) vs. 120 (120, 180)], cognitive symptoms [10 (9, 11) vs. 7 (5, 8)], and doctor-patient communication [10 (9, 11) vs. 7 (7, 8)] were higher in the intervention group compared to the control group (*P* = 0.043, 0.002, <0.001, <0.001). PACIC scores for all dimensions (e.g., patient activation [5 (4, 5) vs. 3 (3, 4)], service system/delivery design [4 (4, 5) vs. 3 (3, 3)], goal setting/tailored care [4 (4, 5) vs. 3 (2, 3)], problem-solving/continuity [4 (4, 5) vs. 3 (2, 3)], and follow-up/collaboration [4 (4, 5) vs. 3 (3, 3)] were higher in the intervention group compared to the control group (all *P* < 0.001). Besides, SBP and DBP levels were lower in the intervention group in comparison to the control group (*P* < 0.001, <0.001). The intervention group reported fewer outpatient visits, emergency visits, and hospitalizations, and hospital services, alongside higher medication adherence and BP control rates compared to the control group (*P* < 0.001, <0.001, 0.027, 0.001).

**Conclusions:**

The E-Coach chronic disease management model combined with the WeChat platform improves self-management ability in hypertension patients, and demonstrates good chronic disease management effectiveness.

## Introduction

Hypertension is a major risk factor for the development of cardiovascular and renal diseases, significantly increasing the likelihood of comorbid conditions such as myocardial infarction, stroke, and heart failure (1). Recent estimates indicate that the global prevalence of hypertension among adults is substantially higher in low- and middle-income countries, affecting approximately 31.5% of the population (1.04 billion people), compared to 28.5% in high-income countries (349 million people) (2). Despite regional differences in hypertension control, the overall global control rate remains unsatisfactory, with less than one-fifth of patients achieving adequate blood pressure (BP) control (3). Therefore, long-term and effective management of hypertension is of great clinical importance.

In recent years, health coaching, particularly electronic coaching (E-Coach), has been increasingly applied in the management of chronic diseases (4). However, previous studies both domestically and internationally have reported certain limitations associated with health coaching interventions, such as prolonged intervention cycles, limited accessibility in remote areas, and high intervention costs (4, 5). Emerging evidence suggests that the application of the E-Coach model in hypertension management has yielded positive outcomes, including improved self-management abilities, medication adherence, and clinical indicators (6). Persell et al. demonstrated that smartphone-based hypertension coaching combined with home BP monitoring significantly improved BP control in patients with uncontrolled hypertension (6). In addition, telemonitoring-supported hypertension management programs have also been shown to improve treatment adherence and BP reduction (7, 8). Recently, Yoon et al. reported in the SMART-BP randomized clinical trial that mobile app–based BP self-monitoring combined with digital feedback effectively improved BP control and self-management behaviors in patients with uncontrolled hypertension (9). In addition, the widespread use of the Internet has played a pivotal role in chronic disease management by providing services such as real-time health monitoring, teleconsultation, bidirectional referral, appointment scheduling, personalized health guidance, and online medication consultation (10, 11). Owing to its convenience and cost-effectiveness, the Internet has become a primary source of health information for patients and the general public (12). In China, WeChat is one of the most popular social media platforms, providing services similar to Facebook and serving as a primary channel for users to access health-related information (12, 13). Previous studies have demonstrated that WeChat-based interventions can improve treatment adherence, self-management behaviors, BP monitoring compliance, and health outcomes in patients with hypertension and other chronic diseases (13–15). However, most existing WeChat-based hypertension interventions mainly focus on health education, medication reminders, and remote monitoring, while relatively few studies have evaluated the combined effect of continuous professional coaching, individualized intervention, and real-time digital follow-up on long-term chronic disease management outcomes.

Of particular note, the “Internet + E-Coach” chronic disease management model represents a more structured and patient-centered management strategy that integrates health coaching with digital technology. This model is grounded in patients' actual health behaviors and is closely aligned with the treatment and rehabilitation process of chronic diseases (16). By leveraging this system, healthcare providers can deliver standardized, high-quality intervention services across different locations, effectively enhancing patients' self-management skills and treatment adherence during home-based rehabilitation (16). Previous research by Xiaojuan Yao demonstrated that this model improved glycemic control, lipid profiles, inflammatory markers, and quality of life in patients with diabetic retinopathy (16), suggesting its potential applicability in other chronic disease populations such as hypertension.

However, evidence regarding the specific effectiveness of the E-Coach chronic disease management model integrated with the WeChat platform in patients with hypertension remains limited, particularly in improving long-term self-management and patient-perceived quality of chronic illness care. To our knowledge, few studies have specifically evaluated this combined strategy in hypertension management using both patient-reported outcomes and objective BP control indicators. Therefore, this study aimed to evaluate the impact of the WeChat-based E-Coach chronic disease management model on self-management ability, Patient Assessment of Chronic Illness Care (PACIC) scores, and BP control in patients with hypertension. The WeChat platform supported health management through real-time monitoring, medication reminders, follow-up communication, and automated abnormal alert notifications, which may help improve treatment adherence and early risk identification. Meanwhile, the E-Coach team provided individualized assessment, face-to-face guidance, and timely intervention based on patients' clinical conditions, thereby potentially enhancing disease awareness, self-efficacy, and long-term management compliance. This study was designed to assess whether this integrated management approach could improve chronic disease management outcomes and provide evidence for optimizing hypertension care.

## Methods

### Ethics statement

The study was approved by the Ethics Committee of Chengdu High-tech Guixi Community Healthcare Center, and written informed consent was obtained from all participants prior to enrollment. Additionally, this study was conducted in accordance with the Declaration of Helsinki and its subsequent amendments. Participants logged into the study platform using their own WeChat accounts and passwords. Patients' personal information was accessible only to authorized medical staff on the platform and the patients themselves. All members of the research team committed not to disclose any patient information outside the scope of the study, thereby fully respecting and protecting participants' privacy.

### Study subjects

All middle-aged and elderly hypertension patients admitted to Chengdu High-tech Guixi Community Healthcare Center from January 2022 to January 2023 were recruited. We present the following article in accordance with the CONSORT reporting checklist.

### Inclusion and exclusion criteria

Inclusion criteria were as follows: (1) Middle-aged and elderly patients diagnosed with essential hypertension according to the 2018 European Society of Hypertension/European Society of Cardiology guidelines. essential hypertension patients were defined as office systolic blood pressure (SBP) were ≥140 mmHg and/or diastolic blood pressure (DBP) ≥ 90 mmHg (17), where middle-aged refers to individuals aged 45–59 years and elderly to those aged 60–74 years. Patients aged 75 years and older were excluded to minimize the influence of comorbid chronic conditions, multi-organ dysfunction, and cognitive decline that may introduce confounding factors and interfere with study outcomes (18, 19); (2) Patients with normal consciousness, cognitive function (Mini-Mental Scale Examination score >27 points), and communication abilities, without any history of mental or psychological disorders; (3) Patients capable of independently using smartphones or computers (confirmed through self-reported information and a brief practical demonstration); (4) Patients with a fixed local residence to facilitate follow-up; (5) Patients covered by national medical insurance; (6) Patients with complete clinical data; and (7) Patients who voluntarily provided informed consent to participate.

Exclusion criteria included the following: (1) Patients with secondary hypertension or malignant hypertension; (2) Patients with severe pulmonary, hepatic, or renal dysfunction; (3) Patients with complex cardiovascular and cerebrovascular diseases, including angina pectoris, severe valvular disease, active rheumatic heart disease, or advanced hypertensive nephropathy; (4) Patients participating in other BP management programs during the study period; (5) Patients with cognitive, speech, or hearing impairments; and (6) Patients with impaired self-care ability or significant limitations in mobility and daily activities (Assessment was conducted using Activities of Daily Living and Instrumental Activities of Daily Living scores).

### Randomization and blinding

Eligible participants were randomly assigned to receive either conventional hypertension management or the E-Coach chronic disease management model based on the WeChat platform. Randomization was conducted by an independent research assistant using a computer-generated random number table, with an equal allocation ratio of 1:1, and the allocation group was stored within sequentially numbered, opaque, sealed envelopes, which were supervised by an investigator who was blinded of the group allocations, follow-up, or data analysis. Due to the significant difference between routine hypertension management and WeChat platform-based E-Coach chronic disease management, participants and care teams could not be blinded to the group assignments. However, they were not involved in the outcome assessments or data analyses. The outcome assessors and data statistical analysts were blinded to the grouping and intervention given.

### Interventions

#### Control group

Patients in the control group received conventional hypertension management. This approach involved a multidisciplinary team led by outpatient physicians, with cardiologists and outpatient nurses as key members. The outpatient nurses assisted cardiologists in patient management, including measuring BP and body mass index (BMI) during clinic visits, providing telephone consultations, and offering routine health education during monthly follow-up visits. The management period lasted for six months.

#### Intervention group

The intervention group received E-Coach chronic disease management model based on the WeChat platform. Descriptions of the functions of the patient-side application system and the medical-side application system are provided in the Supplementary Materials. The intervention was implemented as follows:

Establishment of an E-Coach chronic disease management team based on the WeChat platform: An E-Coach chronic disease management team was established specifically for middle-aged and elderly patients with hypertension. The team included one cardiology specialist (responsible for formulating and timely adjusting treatment plans and addressing disease-related issues), one pharmacist (responsible for medication review and supervision of medication plans), one head nurse (responsible for overall work coordination and supervision of intervention implementation), five specialist nurses (N3 level or above, responsible for implementing intervention measures; N3-level nurses: nurses with more than 5 years of work experience who have passed the N2-level assessment and obtained specialty certification), and one nutritionist, one rehabilitation therapist, and one psychiatrist/psychologist (providing diagnosis, treatment, consultation, and educational services within their respective specialties). Prior to implementation, core members of the E-Coach team, who had been involved in the early development of the management model, conducted comprehensive training sessions. These included eight hours of theoretical instruction covering the E-Coach intervention process, hypertension pharmacotherapy, dietary and emotional management, and exercise guidance, along with two hours of online simulation exercises.

Implementation of E-Coach chronic disease management model: The intervention was primarily carried out by the specialized nurses, with support from the E-Coach team. Patients were the main executors of the management plan, assisted by their family members. The intervention process was structured around the following components:

Contact: Upon enrollment, patients and their families met with specialized nurses to review the study protocol, provide informed consent, and establish communication channels. Patients were added to a WeChat group (“Blood Pressure Care Home”) and subscribed to the “Blood Pressure Manager” official account. Nurses assessed each patient's physiological, psychological, and lifestyle status and jointly developed an individualized management plan.

Observe: Patients monitored their BP and heart rate daily using wearable devices (e.g., Bluetooth Omron BP monitor, model HEM-9200 K, provided free of charge). The nutritionist evaluated dietary habits based on daily meal photos submitted by patients via WeChat and provided personalized online guidance. When necessary, the pharmacist, rehabilitation specialist, and psychiatrist conducted online assessments regarding medication side effects, rehabilitation progress, and psychological status. Additional follow-ups were conducted via WeChat video calls, home visits, or outpatient appointments when needed.

Affirm: Specialized nurses conducted online follow-ups at least once a week during the first month and at least once every two weeks thereafter until the end of the intervention. Follow-ups focused on medication adherence, lifestyle modifications, and risk factor management, while providing encouragement and support to improve patient compliance. Patients were also required to upload data on BP, heart rate, dietary intake, and exercise status regularly.

Clarify: When patients' BP readings exceeded preset thresholds (>=140/90 mmHg), the WeChat platform automatically generated alerts. Red warning signs were used to alert patients and management personnel. Nurses contacted patients with abnormal alerts through online chat or telephone communication and provided personalized guidance, such as medication adjustment, or medical consultation. In cases of poor BP control, inappropriate diet, or insufficient exercise, follow-ups were intensified through voice, video, or face-to-face communication. Dynamic analysis of patient data was used to adjust intervention plans as needed.

Help: Based on patient needs, specialist nurses coordinated with other team members (e.g., psychiatrist, pharmacist) via the WeChat platform to provide structured assessments, treatment plans, and follow-up. The nurses also ensured ongoing communication among team members to facilitate effective patient monitoring and management.

Inspire: Once per month, care services were provided to patients via the WeChat platform to promote positive reinforcement, motivation, and adherence to the management plan.

Nurture: Educational activities and health consultations were regularly conducted through WeChat using multimedia formats, including videos, images, and text, covering topics such as hypertension management knowledge and skills.

Guide: When patients achieved their health goals, positive reinforcement messages or virtual gifts (e.g., flowers) were sent via WeChat to acknowledge their progress. Patients were also encouraged to share their experiences and achievements with family and friends to further strengthen their motivation and sense of purpose.

The intervention period lasted for six months.

#### Outcome measures

The primary outcomes were the Chronic Disease Self-Management Study Measures (CDSMS) and the Patient Assessment of Chronic Illness Care (PACIC). The secondary outcomes were BP values and management effectiveness.

CDSMS (20): The CDSMS was used to assess self-management abilities at baseline and six months after the intervention. The scale comprises three dimensions: physical exercise (stretching, strength training, and aerobic exercise), cognitive symptom management (6 items), and doctor-patient communication (3 items). Physical exercise time was calculated based on patients' responses: not engaging in the activity (0 min/week), <30 min/week (recorded as 15 min/week), 30–59 min/week (45 min/week), 1–3 h/week (120 min/week), and >3 h/week (180 min/week). In the cognitive symptom management (6 items) and doctor - patient communication (3 items) dimensions, each item is scored from zero (never) to five points (always); the maximum score for a single item is 5 points. Each dimension's score is the sum of the scores of its included items. Higher scores indicated better self-management ability. Data collection was performed by trained investigators during outpatient follow-ups, and all questionnaires were completed and returned. (This was also the case for other questionnaires.).

PACIC (21): The PACIC was used to evaluate the effectiveness of chronic disease management at six months post-intervention. The scale consists of five dimensions: patient activation (3 items), delivery system design (3 items), goal setting/tailored care (5 items), problem-solving/continuity (4 items), and follow-up/coordination (5 items). Each item is scored on a 5-point Likert scale, with scores ranging from 1 (“none of the time”) to 5 (“always”). Higher scores indicate better patient-perceived chronic disease care.

BP values: BP values, including SBP and DBP, were measured before the experiment and 6 months after the experiment for both groups. Office BP measurements were taken, with participants instructed not to eat or engage in strenuous exercise 30 min prior to measurement. The average of three BP readings was taken.

Management effectiveness: It was evaluated by comparing the number of outpatient visits, emergency visits, and hospitalizations, and in-person hospital visits (for services such as medical record copying, prescription refills, and insurance review), medication adherence [whether antihypertensive medications were taken as prescribed (Assessment was carried out through pill counting)], and BP control rate. BP control was defined as <130/80 mmHg in patients under 65 years old and <140/90 mmHg in patients aged 65 years or older, in accordance with the guidelines (22).

#### Statistical analysis

Statistical analysis was performed using SPSS 26.0. The Kolmogorov–Smirnov test was used to assess the normality of the distribution. Normally distributed quantitative data were described using mean ± standard deviation ($\bar{x}$ ± s), and comparisons between the two groups were made using the independent samples t-test. Skewed distribution quantitative data were described using median and quartiles [*M* (*P*25, *P*75)], and comparisons between the two groups were conducted using the Mann–Whitney U test, and within-group comparisons were made using the Wilcoxon signed-rank test. Qualitative data were described using numbers and percentages [n (%)] and analyzed using the Chi-squared (*χ*²) test. A *P*-value < 0.05 was considered statistically significant.

## Results

### Demographic data

**Figure 1:**
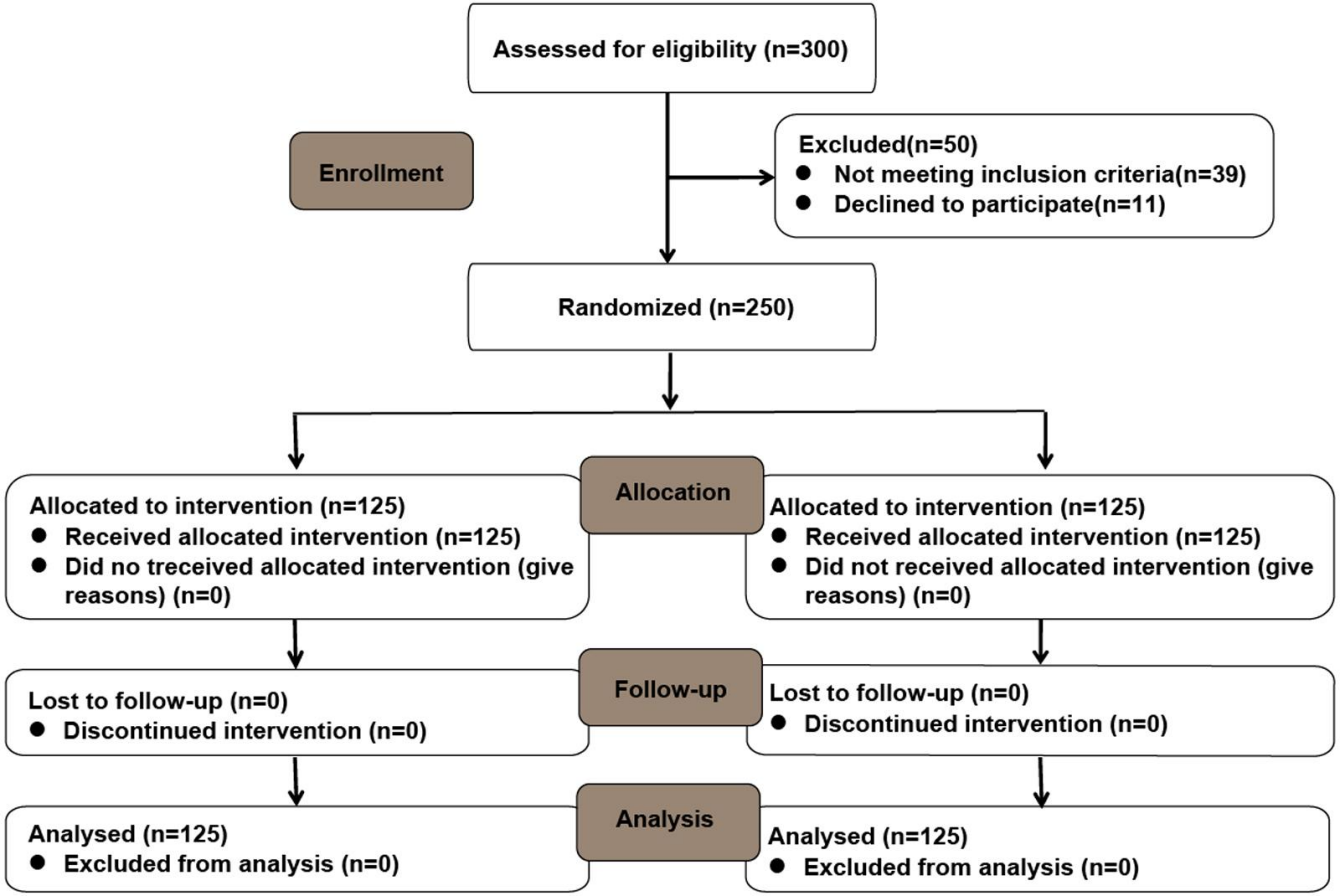
CONSORT flow diagram showing participant flow through the study.

A total of 300 patients were initially recruited. After screening based on the inclusion and exclusion criteria, 250 patients remained for this study. These 250 eligible patients were randomly divided into a control group and an intervention group, with 125 patients in each group (Figure 1). There were no significant differences in general information (sex, age, BMI, duration of hypertension, and educational level) between the two groups, indicating comparability (*P* > 0.05) (Table 1).

**Table 1:** Comparison of general information between the two groups [*n*(%), ($\bar{x}$ ± s), *M* (*P*25, *P*75)].

Indicator	Intervention group (*n* = 125)	Control group (*n* = 125)	*P* (95%CI)	OR/Cohen's d
Gender (*n*, %)			0.307 (0.786∼2.144)	
Male	75 (60%)	67 (53.6%)	-	1.299
Female	50 (40%)	58 (46.4%)	-	
Age (*n*, %)			0.516 (0.711∼1.970)	1.184
45–64 years old	51 (40.8%)	46 (36.8%)	-	
65–74 years old	74 (59.2%)	79 (63.2%)	-	
Body mass index (kg/m^2^)	23.69 ± 2.58	23.86 ± 2.39	0.572 (−0.798∼0.442)	−0.068
Duration of hypertension (years)	10 (7, 14)	10 (8, 12)	0.258	
Education level (*n*, %)			0.294	
Secondary school and below	56 (44.8%)	52 (41.6%)	-	
High school	41 (32.8%)	52 (41.6%)	-	
College degree or above	28 (22.4%)	21 (16.8%)	-	
Grade of hypertension (*n*, %)			0.690	
Grade 1	53 (42.4%)	55 (44%)	-	
Grade 2	45 (36%)	39 (31.2%)	-	
Grade 3	27 (21.6%)	31 (24.8%)	-	
Current number of antihypertensive medications used (*n*, %)			0.311 (0.471∼1,272)	0.774
1 medication	60 (48%)	68 (54.4%)		
≥ 2 medications	65 (52%)	57 (45.6%)		
Comorbidities				
Diabetes mellitus	70 (56%)	62 (49.6%)	0.311 (0.786∼2.127)	1.293
Coronary heart disease	61 (48.8%)	63 (50.4%)	0.800 (0.571∼1.540)	0.938
Hyperlipidemia	74 (59.2%)	66 (52.8%)	0.308 (0.786∼2.140)	1.297

Hypertension grading was performed according to the 2018 ESC/ESH Guidelines for the Management of Arterial Hypertension. The 95% confidence interval (95% CI) refers to an estimated range of values for a population parameter constructed from sample statistics, indicating that, under repeated sampling, the interval would contain the true parameter value in approximately 95% of samples. The odds ratio (OR) is the ratio of the odds of an event occurring in one group to the odds of it occurring in another group and is commonly used to quantify the strength of association between an exposure and an outcome. Cohen's d is a standardized measure of effect size that quantifies the magnitude of the difference between the means of two groups.

### CDSMS scores

**Figure 2:**
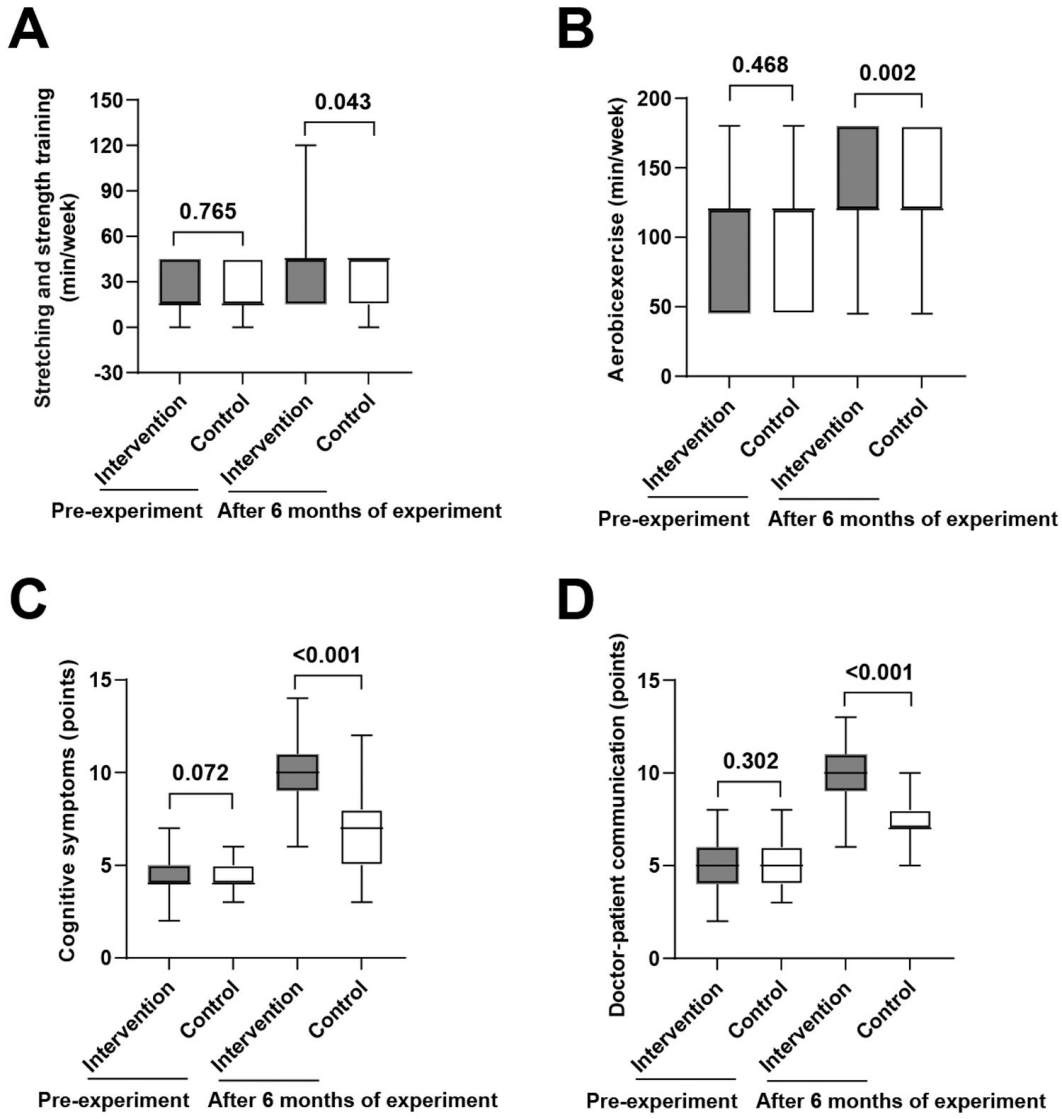
Comparison of CDSMS scores in each dimension between the two groups. **(A)** Stretching and strength training; **(B)** Aerobic exercise; **(C)** Cognitive symptoms; **(D)** Doctor-patient communication.

Before the experiment, there were no significant differences in CDSMS scores between the two groups across all dimensions (stretching and strength training, aerobic exercise, cognitive symptoms, doctor-patient communication) (*P* = 0.765, 0.468, 0.072, 0.302). Six months after the experiment, the CDSMS scores of both groups increased compared to before, with the intervention group scoring higher than the control group across all dimensions (*P* = 0.043, 0.002, < 0.001, < 0.001). Furthermore, the intervention group exhibited significantly greater improvements in all dimensions of the CDSMS compared with the control group (*P* < 0.001, *P* = 0.037, *P* < 0.001, and *P* < 0.001, respectively) (Table 2; Figure 2).

**Table 2:** Comparison of CDSMS scores in each dimension between the two groups [*M* (*P*25, *P*75), min, points].

CDSMS scores	Intervention group (*n* = 125)	Control group (*n* = 125)	*P*
Pre-experiment			
Stretching and strength training	15 (15, 45)	15 (15, 45)	0.765
Aerobic exercise	120 (45, 120)	120 (45, 120)	0.468
Cognitive symptoms	4 (4, 5)	4 (4, 5)	0.072
Doctor-patient communication	5 (4, 6)	5 (4, 6)	0.302
After 6 months of experiment			
Stretching and strength training	45 (15, 45)*	45 (15, 45)*	0.043
Aerobic exercise	120 (120, 180)*	120 (120, 180)*	0.002
Cognitive symptoms	10 (9, 11)*	7 (5, 8)*	<0.001
Doctor-patient communication	10 (9, 11)*	7 (7, 8)*	<0.001
Δ(Stretching and strength training)	0 (0, 18.75)	0 (0, 0)	<0.001
Δ(Aerobic exercise)	60 (0, 75)	60 (0, 75)	0.037
Δ(Cognitive symptoms)	5 (5, 6)	2 (1, 3)	<0.001
Δ(Doctor-patient communication)	6 (5, 6)	2 (2, 2)	<0.001

Compared with the same group before experiment, *P < 0.05. CDSMS, chronic disease self-management study measures. Δ denotes the change from baseline and is calculated as the post-intervention value minus the pre-intervention value.

### PACIC scores

**Figure 3:**
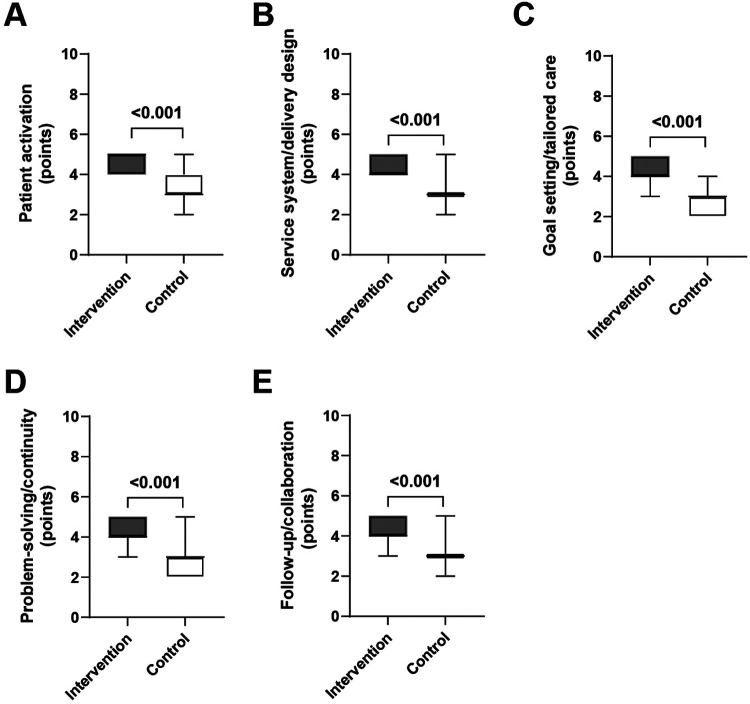
Comparison of PACIC scores in each dimension between the two groups. **(A)** Patient activation; **(B)** Service system/delivery design; **(C)** Goal setting/tailored care; **(D)** Problem-solving/continuity; **(E)** Follow-up/collaboration.

Six months post-experiment, the intervention group achieved higher scores than the control group across all dimensions of the PACIC, including patient activation, service system/delivery design, goal setting/tailored care, problem-solving/continuity, and follow-up/collaboration (*P* < 0.001, < 0.001, < 0.001, < 0.001, < 0.001) (Table 3; Figure 3).

**Table 3:** Comparison of PACIC scores in each dimension between the two groups [*M* (*P*25, *P*75), points].

Dimension	Intervention group (*n* = 125)	Control group (*n* = 125)	*P*
Patient activation	5 (4, 5)	3 (3, 4)	<0.001
Service system/delivery design	4 (4, 5)	3 (3, 3)	<0.001
Goal setting/tailored care	4 (4, 5)	3 (2, 3)	<0.001
Problem-solving/continuity	4 (4, 5)	3 (2, 3)	<0.001
Follow-up/collaboration	4 (4, 5)	3 (3, 3)	<0.001

PACIC, patient assessment of chronic illness care.

### BP values

Prior to the experiment, the median SBP/DBP values in the control group were 156/98 mmHg, while those in the intervention group were 155/96 mmHg, with no significant differences were observed in either SBP or DBP between the two groups (*P* = 0.722, 0.960). Six months post-experiment, the median SBP/DBP values in the control group were 134/83 mmHg, while those in the intervention group were 124/78 mmHg; both groups exhibited a reduction in both SBP and DBP compared to their pre-experiment levels, and the intervention group demonstrated lower BP values compared to the control group (*P* < 0.001, < 0.001) (Table 4).

**Table 4:** Comparison of blood pressure values between the two groups [*M* (*P*25, *P*75), mmHg].

Blood pressure	Intervention group (*n* = 125)	Control group (*n* = 125)	*P*
Pre-experiment			
Systolic blood pressure	155 (148.5, 173)	156 (148, 172)	0.722
Diastolic blood pressure	96 (84, 106.5)	98 (83.50, 106)	0.960
After 6 months of experiment			
Systolic blood pressure	124 (115, 133)*	134 (128, 144.5)*	<0.001
Diastolic blood pressure	78 (74, 84)*	83 (78, 87)*	<0.001

Compared with the same group before experiment.

* *P <* 0.05.

### Management effectiveness

During the experiment period, the intervention group exhibited a lower number of emergency room visits, readmissions, and in-person hospital visits for offline services compared to the control group (*P* < 0.001, < 0.001). Additionally, the intervention group demonstrated higher medication adherence and BP control rates than the control group (*P* = 0.027, 0.001) (Table 5).

**Table 5:** Comparison of management effectiveness between the two groups [*M* (*P*25, *P*75), *n*(%)].

Management projects	Intervention group (*n* = 125)	Control group (*n* = 125)	*P*
Number of outpatient visits, emergency visits, and hospitalizations (times)	3 (2, 3)	4 (3, 4)	<0.001
In-person hospital visits for offline services (times)	4 (3.5, 5)	6 (5, 7)	<0.001
Medication adherence (n)	102 (81.6%)	87 (69.6%)	0.027
Blood Pressure Control (N)	83 (66.4%)	56 (44.8%)	0.001

Medication adherence was assessed using the pill count method by comparing the actual remaining pills with the expected remaining pills to calculate the adherence rate.

## Discussion

The E-Coach chronic disease management model offers a novel solution by integrating internet-based technology with traditional health coaching, effectively leveraging their respective strengths while addressing their limitations (4). This study evaluated the impact of the E-Coach model combined with the WeChat platform on self-management ability and PACIC scores in patients with hypertension. The findings demonstrated that this integrated approach was associated with better self-management ability, improved BP control, and better patient-reported chronic disease care experience.

Specifically, the results showed that patients in the intervention group exhibited significant improvements in all dimensions of the CDSMS compared to the control group. The intervention effectively promoted patient engagement in physical exercise, including stretching, strength training, and aerobic activities, and enhanced cognitive symptom management and doctor-patient communication. These improvements suggest that the E-Coach model not only encourages healthy behaviors but also fosters better communication and interaction between patients and healthcare providers. This is consistent with previous research by Kang et al. (23), which reported that electronic health coaching programs can effectively improve patients' self-efficacy.

In terms of clinical outcomes, the intervention group achieved significantly greater reductions in both systolic and diastolic BP levels after six months compared to the control group. This finding underscores the effectiveness of the E-Coach model in improving BP control, which is critical for reducing the risk of hypertension-related complications. This result is consistent with recent hypertension-specific digital intervention studies. Yoon et al. reported in the SMART-BP randomized clinical trial that mobile app–based BP self-monitoring combined with digital feedback led to greater systolic BP reduction and improved medication adherence in patients with uncontrolled hypertension (9). Similarly, Sun et al. developed a WeChat-based behavioral digital intervention for patients with hypertension and reported improvements in BP control and healthy behavior adherence (24). Wang et al. also found that a WeChat-based multimodal digital intervention improved BP control rates and reduced BP levels compared with usual care (25). These findings suggest that digital platforms may enhance hypertension management by strengthening BP monitoring, medication adherence, feedback efficiency, and patient engagement. Additionally, the application of the Internet + E-Coach model in diabetic retinopathy patients has been shown to improve disease awareness and stabilize metabolic indicators such as blood glucose and lipid levels (16).

Furthermore, the intervention group demonstrated superior outcomes in overall management effectiveness. Compared with the control group, patients receiving the E-Coach intervention had fewer outpatient visits, emergency visits, and hospitalizations, and in-person healthcare service requirements, suggesting a potential reduction in healthcare resource utilization. Medication adherence and BP control rates were also significantly higher in the intervention group, indicating improved treatment compliance and better disease management. These findings are broadly consistent with recent evidence showing that digital health interventions, including mobile health, app-based BP self-monitoring, digital feedback, and WeChat-based management, can improve BP control, adherence, and self-management behaviors in patients with hypertension. A recent systematic review also suggested that digital health interventions may be effective in reducing systolic BP and improving BP control among adults with hypertension, although heterogeneity across intervention models remains considerable (26). Evidence further supports that E-Coach interventions and WeChat-based health management measures can enhance medication adherence and patients' willingness to seek medical care (4, 13). Additionally, the incidence of adverse events was lower in the intervention group, likely attributable to the individualized and continuous care provided through the E-Coach model, which facilitated early identification and management of health risks. The PACIC scores after six months also indicated better patient-perceived quality of chronic illness care in the intervention group. It should be noted that PACIC does not simply measure patient satisfaction; rather, it reflects patients' perceptions of the organization, coordination, patient-centeredness, and continuity of chronic illness care. This may be attributed to the program's emphasis on patient-centered care, including individualized goal setting, tailored care plans, problem-solving strategies, and consistent follow-up support. However, these improvements should be interpreted cautiously. The observed benefits may not be entirely attributable to the E-Coach model itself, but may also reflect increased contact intensity, closer monitoring, and greater attention received by patients in the intervention group. In addition, providing a free Bluetooth BP monitor to the intervention group may have increased measurement frequency, patient engagement, and possible socioeconomic incentives, which could partly confound the intervention effect.

However, several limitations of this study should be acknowledged. First, some outcomes, including CDSMS and PACIC scores, were based on self-reported questionnaires and may therefore be influenced by subjective assessment, recall bias, and reporting bias. Because participants and care teams could not be blinded due to the nature of the intervention, performance bias and reporting bias could not be completely avoided. In addition, as this study was not blinded, there is an inherent risk that participants in the intervention group may have been more likely to report favorable outcomes, which should be considered when interpreting the findings. Second, although the sample size was adequate for preliminary analysis, no formal *a priori* sample size calculation was performed before the study, and multiple outcomes and sub-dimensions were analyzed, which may have increased the risk of false-positive findings. Moreover, no formal correction for multiple comparisons was conducted, which may further increase the possibility of type I error. Third, despite efforts to minimize contamination, unequal attention between groups and possible group contamination could not be completely excluded. In addition, although the intervention was delivered through the WeChat platform, detailed data regarding patients' adherence to platform use, engagement frequency, and changes in utilization over time were not systematically collected. Therefore, the relationship between intervention adherence and clinical outcomes could not be evaluated, which may have limited a more comprehensive understanding of the mechanisms underlying the observed effects. Fourth, the strict inclusion criteria, such as independent smartphone use, local residence, national medical insurance coverage, and exclusion of patients aged ≥ 75 years, may have limited the generalizability of the findings, particularly for older adults and patients with lower digital literacy. In addition, patients with severe comorbidities or cognitive impairment were excluded to improve intervention adherence and reduce potential confounding factors; however, this may further limit the applicability of the findings to older hypertensive populations, who are important target groups for community-based digital health interventions. Furthermore, no subgroup analyses based on age, educational level, or digital literacy were performed, and therefore potential differences in intervention effectiveness among different patient populations could not be fully evaluated. In addition, this was a single-center study conducted in one community healthcare center, which may reduce the external validity of the results. Moreover, although the intervention group achieved greater reductions in blood pressure, both groups showed significant BP reductions from baseline, suggesting that many participants may have had previously suboptimally controlled hypertension or may have benefited from increased clinical attention during the study period. Therefore, the intervention effect should be interpreted with caution. Finally, the follow-up period was limited to six months, and no formal cost-effectiveness analysis was performed; therefore, the long-term sustainability, scalability, and practical applicability of this intervention still require further investigation. Furthermore, the conversion of categorical variables such as weekly exercise duration into single numerical values may have introduced some degree of measurement bias and reduced the variability of the original data, which could potentially affect the accuracy of the statistical analysis.

Given these limitations, future research should focus on several areas. First, large-scale, multicenter studies are needed to further validate the effectiveness of the E-Coach chronic disease management model combined with the WeChat platform. Second, the applicability of this model in the management of other chronic diseases warrants further exploration. Third, future studies should include formal sample size calculation, more rigorous missing-data strategies, and clearer intervention fidelity reporting. In addition, long-term follow-up and formal cost-effectiveness analyses are needed before recommending large-scale integration of this model into routine clinical practice. Evaluation of staffing burden, implementation feasibility, and scalability should also be considered, particularly for primary healthcare settings and resource-limited communities.

In summary, the E-Coach chronic disease management model integrated with the WeChat platform was associated with improvements in self-management ability, BP control, and patient-reported chronic disease care experience in patients with hypertension. The potential value of this model lies in its combination of personalized care, real-time feedback, and continuous support. However, given the limitations related to subjective assessment, possible confounding factors, restricted generalizability, and the lack of formal cost-effectiveness evaluation, these findings should be interpreted with caution. Further high-quality multicenter studies are needed before broad implementation in routine clinical practice can be strongly recommended.

## Data Availability

The original contributions presented in the study are included in the article/Supplementary Material, further inquiries can be directed to the corresponding author.
