# Supplementary material for: Impact of E-Coach chronic disease management model combined with the WeChat platform on self-management ability in hypertension patients
Source: Front Cardiovasc Med. 2026 Jul 6;13:1707441. doi: 10.3389/fcvm.2026.1707441 (PMC13381742; doi:10.3389/fcvm.2026.1707441)
Supplement: Supplementary file 1 [file Table1.pdf]

Functional description of the patient-side application system

Interface	Main Module	Submodule	Function Description
Login Interface	Contract Application	Registration	Register using ID card number and gesture password.
		Verification Code	Receive a verification code via the registered mobile phone number to complete registration.
		Agreement	
	Login	Login	Log in using the mobile phone number and gesture password.
		Remember Account	Save the login account information.
		Forgot Password	Retrieve or reset the password.
Homepage	My Information		Includes patients' basic information, contact information, and contract validity period.
	My Goals		Includes BP control targets, lifestyle management goals, and regular monitoring tasks.
	Daily Records	Blood Pressure Records	Upload measurement date, time, measurement method, and BP values.
		Examination Reports	Automatically retrieved from the hospital information system or manually uploaded.
		Dietary Records	Upload daily dietary intake information.
		Exercise Records	Upload exercise type, frequency, average duration of each session, and heart rate.
		Emotional Records	Upload daily psychological/emotional status.
		Medication Records	Upload daily medication-taking status.
		Data Query	Review health records, with automatic links to health tips and graphical displays.
	Health Assistant		View personalized health information and health education materials.
	Medication Plan		Add or remove medications and set medication reminders as needed.
	Mid-term Evaluation		Evaluate disease-related risk factors every two weeks.
	Health Information		Regularly send reminders and health education information to patients.
Contact Doctor	Medical staff and dedicated administrators provide online consultation and communication services at fixed times every day.		

Functional description of the medical-side application system

Main Menu	Main Module	Submodule	Function Description
Login Interface	Login		Log in using the physician's account and password.
Homepage	System Management	Team Management	Differentiate team names and codes and manage the addition or removal of physicians.
		Login Log Query	
		Group Management	Create and manage custom groups conveniently.
		Chat Templates	Create custom chat template titles and content.
	Contract Management	Contract Application	Review and approve or reject applications.
		Contracted Patients	View patient-related information and terminate contracts if necessary.
	Data Query		Query and export patient-uploaded monitoring data by time and enable bidirectional referral.
	Data Alerts		Automatically generate alerts for abnormal BP, non-compliance with lifestyle recommendations, missed medication, failure to log into the platform, or missing records, and send reminders to patients.
	Medication Review		Pharmacists review medication prescriptions and provide feedback on inappropriate medication use, which is accessible only to hospital physicians.
	Online Chat		(1) Physicians can communicate online with their assigned patients; (2) designated online service staff are granted permission to communicate with all users during fixed daily service hours.
	Training Courses		Patient-oriented educational courses maintained by the hospital, supporting uploads of Word documents, PowerPoint presentations, images, videos, and other materials.
	Data Maintenance		Add, modify, or delete medications and revise nursing/intervention plans.
